# Investigating the efficiency of *oak* powder as a new natural coagulant for eliminating polystyrene microplastics from aqueous solutions

**DOI:** 10.1038/s41598-023-47849-4

**Published:** 2023-11-21

**Authors:** Afsaneh Esmaeili Nasrabadi, Mohaddeseh Zahmatkesh Anbarani, Ziaeddin Bonyadi

**Affiliations:** 1 Student Research Committee, Mashhad University of Medical Sciences, Mashhad, Iran; 2 Department of Environmental Health Engineering, School of Health, Mashhad University of Medical Sciences, Mashhad, Iran

**Keywords:** Biotechnology, Chemistry, Engineering

## Abstract

Polystyrene (PS) is a commonly used plastic material in disposable containers. However, it readily breaks down into microplastic particles when exposed to water environments. In this research, oak powder was used as a natural, inexpensive, and eco-friendly coagulant. The present study aims to determine the effectiveness of oak powder in removing PS from aquatic environments. The Box-Behnken model (BBD) was used to determine the optimal conditions for removal. The removal efficiency was evaluated for various parameters including PS concentration (100–900 mg/L), pH (4–10), contact time (10–40 min), and *oak* dosage (100–400 mg/L). The maximum removal of PS microplastics (89.1%) was achieved by using an *oak* dose of 250 mg/L, a PS concentration of 900 mg/L, a contact time of 40 min, and a pH of 7. These results suggest that *oak* powder can effectively remove PS microplastics through surface adsorption and charge neutralization mechanisms, likely due to the presence of tannin compounds. Based on the results obtained, it has been found that the natural coagulant derived from *oak* has the potential to effectively compete with harmful chemical coagulants in removing microplastics from aqueous solutions.

## Introduction

The demand for plastic is constantly increasing due to its low cost, flexibility, resistance to oxidation, and high durability. Recently, plastic production worldwide has increased dramatically. It is estimated that the production of plastics will increase from 1.5 million tons in 1950 to 445.25 million tons in 2025^1^. Due to the low rate of recycling, 90% of plastics end up being discarded into the natural environment, especially in aquatic ecosystems^2^. Plastics can be transferred from terrestrial ecosystems to aquatic ecosystems due to their hydrophobic properties and their physical and chemical durability. Pollutants are present in various environments, including fresh surface water and sediments, sea surface water and seabed, underground water, soil, and even the atmosphere^3^. The decomposition of plastic in the environment, caused by physical destruction, ultraviolet rays, weathering, and oxidation results in the formation of tiny particles known as microplastics, which are less than 5 mm in size^4^.

Microscopic plastic particles and their widespread distribution in aquatic environments are a global issue of significant environmental concern. The significance of microplastic particles at a small scale and their extensive dispersion in aquatic ecosystems has recently been acknowledged as a worldwide problem that is causing growing environmental apprehension. Microplastics can easily adsorb hazardous substances such as polycyclic aromatic hydrocarbon compounds (PAHs), cyanide^5^, and pesticides^6^, as well as rare earth metal elements, due to their large specific surface area and hydrophobic surface properties. Microplastics present in water bodies can be mistaken for food by aquatic organisms, leading to various issues such as intestinal blockage. These minuscule plastic particles can infiltrate the food chain and ultimately pose a threat to human health^7^. Research has demonstrated that microplastics can penetrate the cells of various body organs through biological membranes and interfere with the proper functioning of the digestive, respiratory, nervous, and endocrine systems when ingested or inhaled^8^. Microplastics pose a range of ecological and health concerns. They can be ingested by a wide range of organisms, including fish, birds, marine mammals, and invertebrates. This ingestion can cause physical harm. Additionally, microplastics can adsorb and transport toxic chemicals, such as persistent organic pollutants and heavy metals. This raises concerns about their potential to bioaccumulate and enter the food chain, ultimately impacting human health^7,9^. Since seafood is an important source of protein and vitamins, by consuming seafood, microplastic particles enter the human body through the food chain^10^.

Microplastics exist in various types of polyethylene, polypropylene, polystyrene, and polycarbonates in aquatic environments^11^. PS is the second most prevalent microplastic in fresh water, soil sediments, and coastal ecosystems^12^. PS is one of the most widely used plastic materials in disposable vessels, which easily decomposes into microplastic particles in water environments^13^.

Recently, treatment technologies such as membrane bioreactors^14^, rapid sand filtration^15^, and coagulation and flocculation processes^16,17^ have been increasingly utilized to eliminate microplastics^18^. The coagulation and flocculation process is widely used in water and wastewater treatment systems due to its simplicity and efficiency. The coagulation process operates primarily by neutralizing the negative charges of colloidal particles, leading to their destabilization and eventual sedimentation^19^. Coagulants that are mainly used in purification include aluminum sulfate (alum), iron chloride, and synthetic organic polymers^19,20^. To eliminate emerging pollutants from aquatic environments, researchers are seeking environmentally friendly and biodegradable methods. The use of methods based on natural coagulants, such as chitosan and tannins found in the skin and fruits of trees, is effective in purifying water and wastewater, particularly in separating suspended particles. Natural coagulants, such as chitosan and tannins found in bark and tree fruits, facilitate particle aggregation and enable the separation of suspended particles. Tannin is a naturally occurring cationic polymer with a low molecular weight that is derived from plants. This natural coagulant is used in the treatment of water and wastewater^21^.

An *oak* is a tree or shrub belonging to the genus Quercus in the beech family, Fagaceae. More than 500 species of *oak*, including both trees and shrubs, grow in various regions around the world. The vegetation in the western and northern forests of Iran is abundant with *oak* trees. People in various regions of the world have been utilizing the fruit of the *oak* tree for numerous years^22^. *Oak* fruits contain significant amounts of phenolic compounds and tannins^23^. Tannins are polyphenolic compounds with a high molecular weight. They are classified into different types based on their chemical structure and properties^24^. *Oak* fruit contains 41 phenolic and polyphenolic compounds, including hydroxybenzoic acid and its derivatives, gallotannins, phenolic glycosides, ellagitannins, and ellagic acid and its derivatives^23^. *Oak* residues are a highly effective organic coagulant in water treatment processes due to their abundance, low cost, compatibility with the environment, and high tannin content^25^. They have been shown to effectively remove various pollutants from water environments^26^. Zhu et al.^27^ removed microplastics made of polyethylene and polystyrene using ferric chloride and polyaluminum chloride. Rajala et al.^28^ investigated the removal of microplastics from secondary effluents using iron, aluminum, and polyamine-based chemicals.

This study focuses on evaluating the effectiveness of* oak* powder as a natural coagulant for removing PS MPs from aqueous solutions. Various parameters, such as *aok* dose, PS concentration, contact time, and pH, were examined to optimize the coagulation process and gain a better understanding of its mechanisms. The effectiveness of PS removal was assessed by measuring the levels of polystyrene before and after treatment with *oak* powder. It is important to note that this study exclusively investigates PS MPs and does not include other types. Additionally, the study is limited to aqueous solutions and does not involve non-aqueous or organic solvents.

## Materials and methods

### Chemicals and reagents

PS granules were purchased from Pishgaman Plastic Company in Iran, and the necessary chemicals were obtained from Merck in Germany.

### Preparation of *oak* coagulant

The discarded *oak* powder were collected from western Iran. The discarded powder were crushed, washed with tap water, and then dried in an oven at 60 °C for 24 h. The resulting dry material was ground into a powder with a fineness of less than 100 mesh.

### Preparation of PS microplastic

The PS granules underwent a washing process with 0.1 M HCl, followed by drying at 60 °C for 24 h. They were then crushed using a grinder and meshed into sizes of 300 µm, 425 µm, and 850 µm. PS particles were stored in a dark and sealed container. Exposure to light may cause decomposition and alterations in the physical properties and molecular weight of the substance. This causes errors in the test results.

### Characteristics and measurements

The characteristics of *oak* and PS particles were investigated by FTIR, FESEM, and EDX tests. FTIR spectroscopy was utilized to measure the surface functional groups of *oak* and PS, as well as the functional groups present after the experiment and removal process. The FESEM test was used to determine the surface morphology of *oak* and PS. Additionally, EDX test was utilized to determine the elemental composition the particles.

### Fourier Transform Infrared Spectrometer (FTIR)

FTIR analysis was utilized to compare and identify the chemical compounds, bonds, and functional groups present before and after the synthesis of the adsorbent^29^. This test was conducted using a PerkinElmer spectrometer (FT-IR/NIR Frontier).

### Field emission scanning electron microscope (FESEM)

FESEM can be used to observe changes in surface morphology of flocs composed of *oak* particles and microplastics^9^. FESEM was conducted using a Supra 55 electron microscope manufactured by Carl Zeiss in Germany.

### Energy dispersive X-ray (EDX)

EDX method is used to evaluate the elemental composition of PS samples before and after the removal process^30^. For this purpose, we used the Oxford Instruments SEM model JEOL-JSM-5600.

### Design of experiments

The efficiency of removal was evaluated based on the initial PS concentrations, pH, contact time, and *oak* dosage (refer to Table 1). All experiments were conducted in a 250 mL flask containing 200 mL of the reaction mixture.

**Table 1:** Parameters required for the removal of PS microplastics.

Factor	Code	Variable level		
		− 1	0	+ 1
PS Conc. (mg/L)	A	100	500	900
*Oak* dose (mg/L)	B	100	250	400
pH	C	4	7	10
Time (min)	D	10	25	40

The size of the PS particles was less than 100 µm. All tests were conducted at room temperature (25 °C). A jar test apparatus was utilized to conduct the tests. A 200 cc of the reaction solution was agitated using a Jar test apparatus at 400 rpm for 1 min to ensure direct contact between PS particles and *oak* particles. The speed was then reduced to 100 rpm for 15 min to facilitate the formation of larger flocs. After the reaction time ended, the suspension of PS and *oak* was poured into the Imhoff funnel. The flocs that had formed were allowed to settle under static conditions for 30 min. Afterward, the supernatant was collected and filtered through a 45 µm Whatman filter paper. The filtered solution was then dried in an oven at 60 °C for 24 h. The PS removal efficiency was determined using the weighing method and the following formula:

1 $$ {\text{R}} \left( \% \right)\; = \;\frac{{{\text{M}}_{1} - {\text{M}}_{2} }}{{{\text{M}}_{1} }} \times 100\% $$

In this formula, M1 represents the initial weight of PS before the removal process, while M2 is the weight of PS on the Whatman filter after the removal process.

### Modeling the removal of PS

The present study was designed using BBD model to optimize the process of removing PS microplastics. BBD is a popular RSM design that offers several advantages. A significant advantage of this design is its ability to achieve efficient testing by requiring fewer test runs compared to other designs, such as the full factorial design. This decrease in the number of runs makes BBD a practical choice when resources are limited. BBD excels in studying quadratic response surfaces and enables researchers to efficiently analyze and estimate main effects, interactions, and quadratic effects^31^. By capturing these fundamental factors, the BBD model facilitates a comprehensive understanding of complex systems and their non-linear relationships. The quadratic model used by RSM is expressed as the following equation:

2 $$ {\text{Y}} = \beta 0 + \mathop \sum \limits_{i = 1}^{k} \beta_{i} x_{i} + \mathop \sum \limits_{i = 1}^{k} \beta_{ii} x_{i}^{2} + \mathop \sum \limits_{1 \le i \le j}^{k} \beta_{ij} x_{i} x_{j } $$

where Y, β_0_, β_i_, βii, β_ij_, and X_i_ or X_j_ represent the predicted response, constant coefficient, regression coefficients for linear impacts, quadratic coefficients, interaction coefficients, and coded values of the parameters, respectively^31^.

## Results and discussion

### Characterization

**Figure 1:**
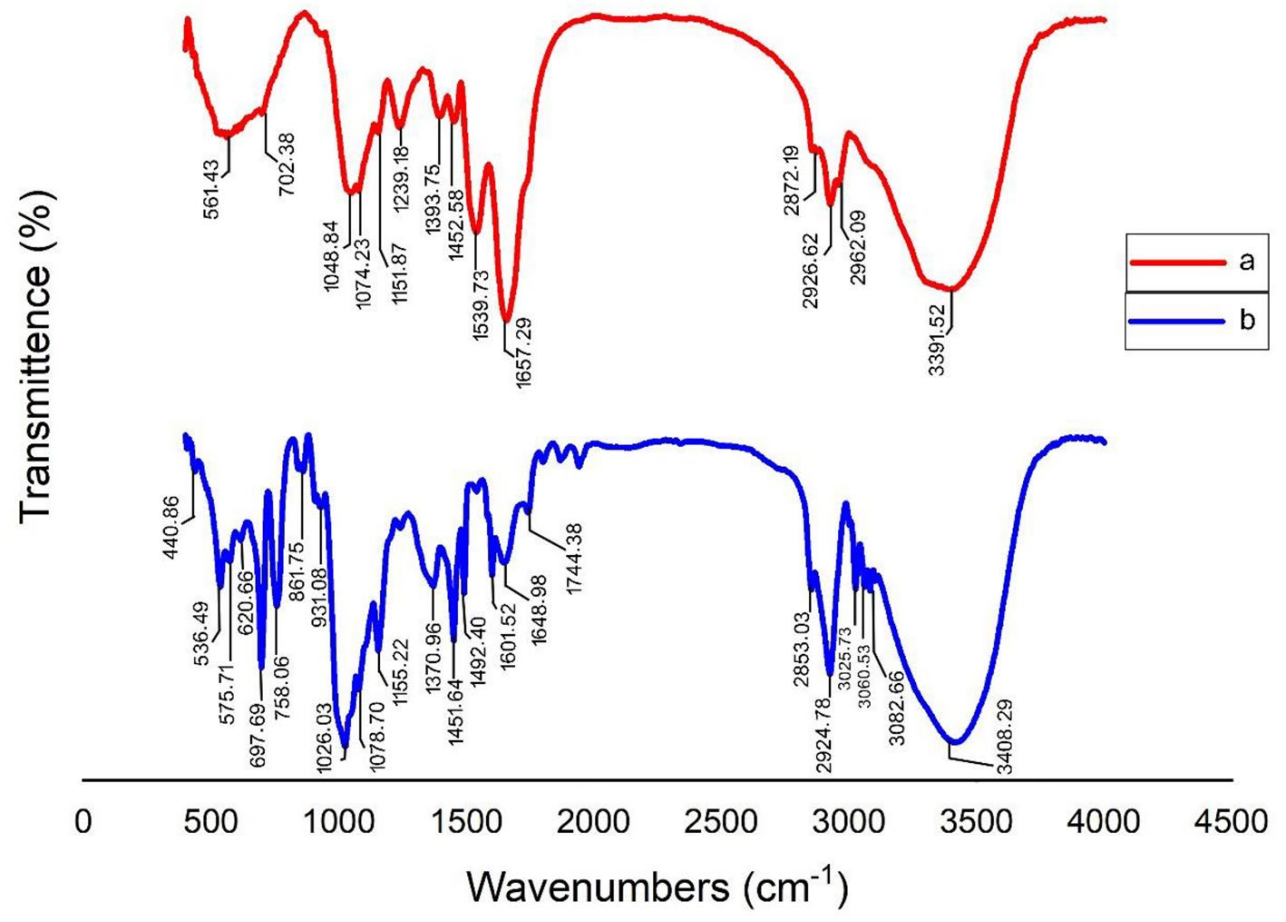
FTIR Spectra of (**a**) before and (**b**) after PS removal.

Figure 1a depicts the FT-IR spectra of PS particles before and after the removal process. The bands observed at 3391.52 and 2926.62 cm^−1^ are likely related to the H–OH vibration of water molecules, stretching of C–H bonds, and bending vibrations, respectively. The peak at 1657.29 cm^−1^ is attributed to the C=O stretching band. During the industrial manufacturing process of PS, these particles combine with oxygen and moisture at high temperatures to form carbon–oxygen (C=O) bonds. The C=O functional group connects PS particles to *oak* powder via hydrogen bonds. It is therefore conceivable to remove PS particles using this method^32^. The peak at approximately 702.38 cm^−1^ indicates the presence of benzene rings in PS^27^. Figure 1b displays the FT-IR spectra following the coagulation process. Compared to the PS spectrum before removal, the peak corresponding to H–OH shifted to 3408.29 cm^−1^, indicating the hydrolysis of the coagulant^33^. Similarly, the intensity of the peak associated with the C=O functional group has changed to 1648.98 cm^−1^. Furthermore, the peak at 2924.78 cm^−1^, which corresponds to C–H groups, did not exhibit significant changes compared to its state prior to removal. Generally, it can be said that the displacement of the peaks is related to the formation of new bonds between the functional groups present in *oak* powder, acting as coagulant particles, and PS.

**Figure 2:**
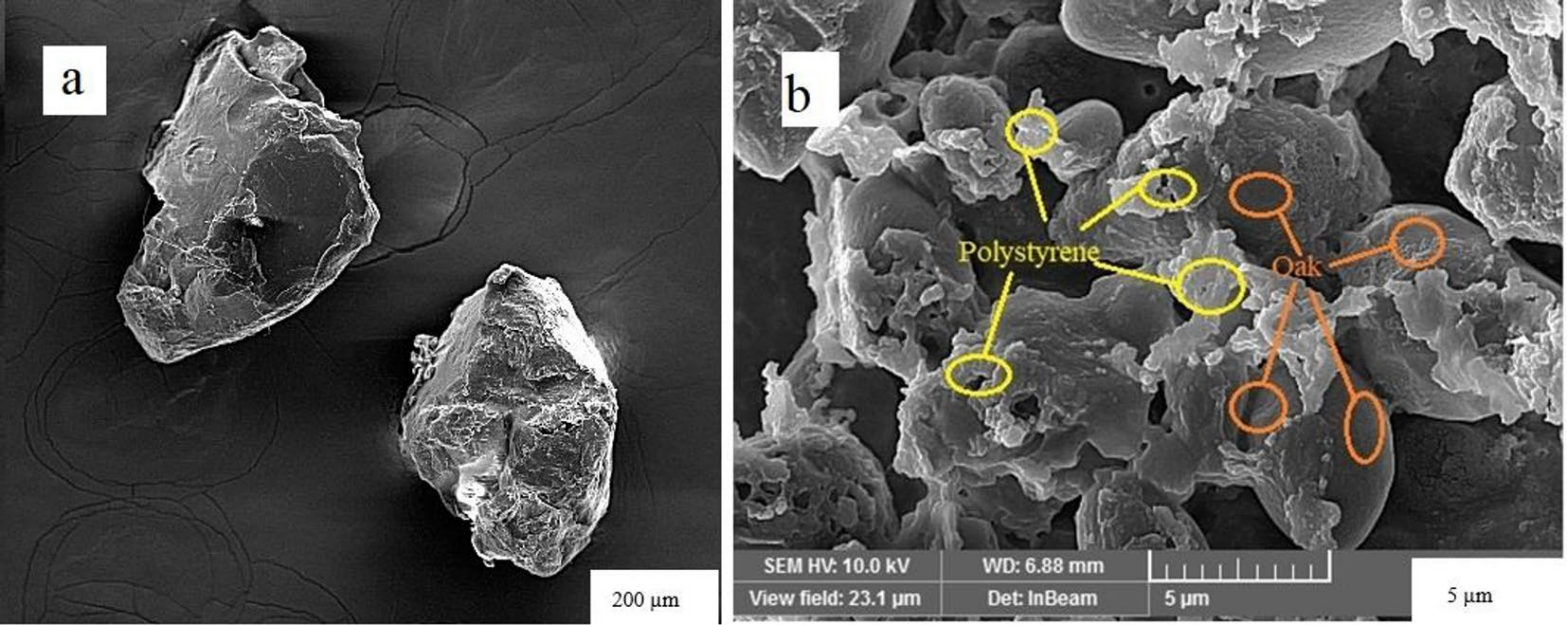
FESEM of (**a**) PS and (**b**) flocs of PS and *oak* powder.

FESEM images of PS and the flocs of PS and *oak* powder are presented in Fig. 2. From Fig. 2a, cracks can be seen on the surface of these particles, which increases their contact surface with the *oak* particles. ​According to the findings presented in Fig. 2b, PS particles accumulate on the surface of *oak* particles. Given that the surface charges of *oak* and microplastic particles are often opposite, it can be inferred that a bridge forms between them, resulting in a charge neutralization process that leads to the formation of large flocs^34^. As can be seen from the FASEM image, the connection between *oak* and microplastic particles is well-established. After the removal process, the connection between the *oak* particles and the rough surfaces of the PS particles has resulted in the formation of larger flocs, thereby increasing the sedimentation rate^32,35^. This finding demonstrates that *oak* powder are effective as coagulants.

**Figure 3:**
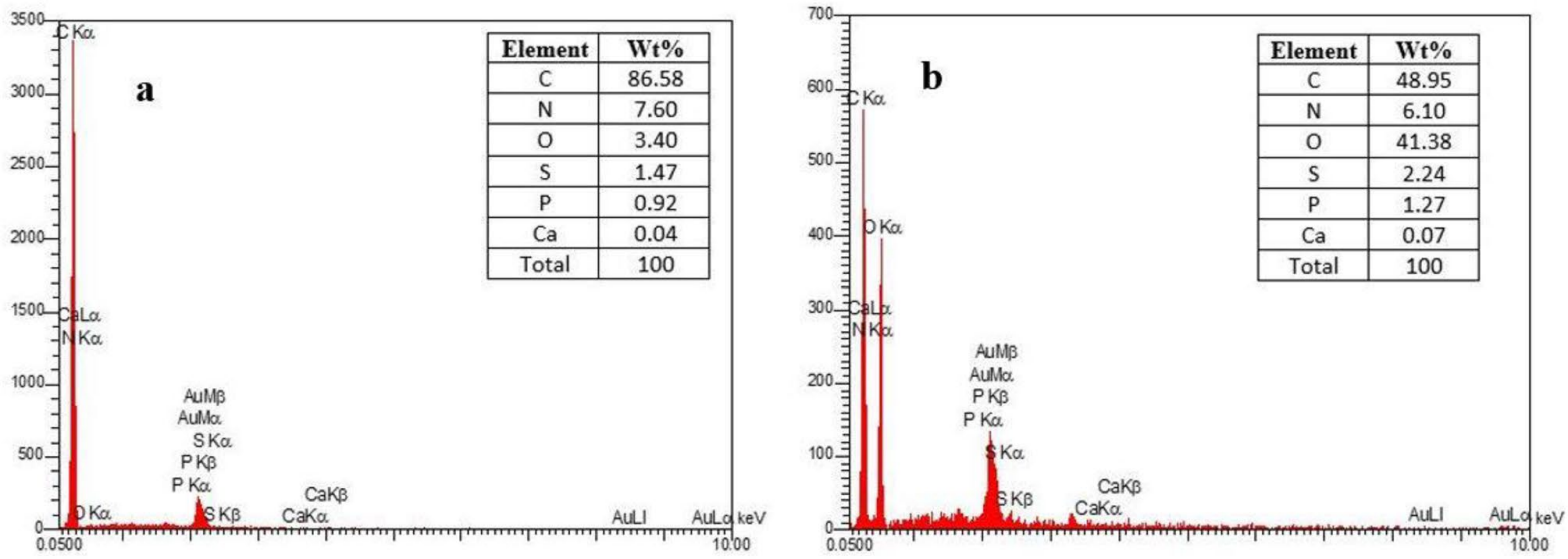
EDX spectrum of PS (**a**) before and (**b**) after removal.

Figure 3 shows the EDX spectrum before and after PS removal. Each peak in the EDX spectrum corresponds to a specific energy level that represents a particular element. The concentration of each element affects the intensity of the peak^36^. Based on the EDX image (Fig. 3a), the levels of carbon, nitrogen, oxygen, sulfur, phosphorus, and calcium elements in PS are displayed. Figure 3b illustrates the changes in element values after binding *oak* particles to PS. The carbon content depicted in Fig. 3a indicates that PS particles exhibit a strong affinity towards binding to the surface of *oak* particles^37^. According to a study, microplastics have been found to adhere to grain surfaces due to their high carbon content^38^.

According to Fig. 3b, *oak* and microplastic flocs contain 41.38% oxygen and 48.95% carbon. Other elements, including calcium, phosphorus, sulfur, and nitrogen, make up a small percentage of this composition. The high oxygen and carbon content in the flocs derived from *oak* and microplastics indicates that *oak* powder, which act as a natural coagulant, are highly effective in eliminating pollutants from aqueous solutions. High carbon content can cause *oak* particles to adhere to microplastics and form flocs, thereby increasing the efficiency of PS removal^39^.

### Response model

In this study, we investigated the effect of *oak* powder on the removal of PS from aqueous solutions. Table 2 displays the removal of PS by *oak* powder.

**Table 2:** BBD matrix for the removal of PS by *oak* powder.

Run No	Coded variable				Removal (%)	Run No	Coded variable				Removal (%)
	A	B	C	D			A	B	C	D	
1	− 1	0	1	0	7.23	16	0	0	0	0	45.5
2	0	1	0	− 1	45.4	17	− 1	0	− 1	0	25.25
3	0	1	− 1	0	53.4	18	0	0	0	0	43.5
4	0	0	1	− 1	80.7	19	− 1	0	0	− 1	27.8
5	0	0	0	0	49.1	20	0	0	− 1	1	83.2
6	1	− 1	0	0	43.8	21	1	0	1	0	78.7
**7**	0	0	1	1	76.2	22	0	1	0	1	65.8
8	0	− 1	− 1	0	45.3	23	1	0	0	− 1	57.9
9	− 1	− 1	0	0	3.26	24	− 1	1	0	0	15.6
10	0	− 1	0	1	55.2	25	0	0	− 1	− 1	71.5
11	0	1	1	0	62.6	26	0	0	0	0	54.8
12	1	1	0	0	44.5	27	0	− 1	1	0	35.4
13	0	0	0	0	38.3	28	1	0	− 1	0	65.6
14	0	− 1	0	− 1	52.4	29	− 1	0	0	1	25.6
15	1	0	0	1	89.1						

According to Table 2, the PS removal efficiency ranged from 3.26 to 89.1%. To select the best model, we statistically evaluated the experimental findings for linear, 2FI, quadratic, and cubic models. Table 3 represents the evaluation of the statistical adequacy of the models.

**Table 3:** Statistical adequacy evaluation of models.

Source	Sequential p-value	Lack of Fit p-value	Adjusted R^2^	Predicted R^2^
Linear	0.0019	0.0233	0.4117	0.2175
2FI	0.8788	0.0156	0.3048	− 0.4486
Quadratic	< 0.0001	0.4537	0.9093	0.7855
Cubic	0.2787	0.6146	0.934	0.5424

Table 4 displays the coefficients of the quadratic model for PS removal using *oak* particles. The quadratic model (QM) was fitted to the experimental data as shown in Table 4.

**Table 4:** Estimation coefficients for the quadratic model of PS removal using *oak* powder.

Factor	Coefficient estimate	df	Standard error	95% CI low	95% CI high	VIF
Intercept	46.24	1	2.98	39.84	52.64	
A-Conc	22.91	1	1.93	18.77	27.04	1
B-Dose	4.33	1	1.93	0.1975	8.46	1
C-pH	− 0.285	1	1.93	− 4.42	3.85	1
D- Time	4.95	1	1.93	0.8192	9.08	1
AB	− 2.91	1	3.34	− 10.06	4.24	1
AC	7.78	1	3.34	0.6253	14.93	1
AD	8.35	1	3.34	1.2	15.5	1
BC	4.78	1	3.34	− 2.38	11.93	1
BD	4.4	1	3.34	− 2.75	11.55	1
CD	− 4.05	1	3.34	− 11.2	3.1	1
A^2^	− 13.05	1	2.62	− 18.67	− 7.44	1.08
B^2^	− 8.26	1	2.62	− 13.88	− 2.65	1.08
C^2^	12.04	1	2.62	6.42	17.66	1.08
D^2^	17.75	1	2.62	12.13	23.37	1.08

Based on Table 4, the QM calculation of PS removal, according to the coded parameters, is presented in Eq. (3):

3 $$ \begin{aligned} Removal \% & \; = \; 46.24 + 22.91A + 4.33B {-} 0.28C + 4.95D {-} 2.91AB + 7.78AC + 8.35AD \\ & \;\;\; + 4.78BC + 4.40BD - 4.05CD {-} 13.05A2 {-} 8.26B2 + 12.04C2 + 17.75D2 \\ \end{aligned} $$

According to Eq. (3), each model comprises of two fixed and variable components. The removal rate was predicted to be 46.24% based on the various test variables. The coded parameters A, B, C, and D had coefficients of + 22.91, + 4.33, − 0.285, and + 4.95, respectively. The concentration of PS (code A) had the greatest effect on removal, with a coefficient of + 22.24. The interaction effect with the highest coefficient was related to AD, with a value of + 8.35. The highest square effect was correlated with D2, with a coefficient of + 17.75. Table 5 illustrates the analysis of variance (ANOVA) for the QM of PS removal by *oak* powder.

**Table 5:** ANOVA results for the quadratic model of PS removal by *oak* powder.

	Sum of squares	df	Mean Square	F-value	p-value
Model	13,125.77	14	937.56	21.06	< 0.0001
A- PS	6295.67	1	6295.67	141.44	< 0.0001
B- Dose	224.81	1	224.81	5.05	0.0413
C- pH	0.9747	1	0.9747	0.0219	0.8845
D- Time	294.03	1	294.03	6.61	0.0222
AB	33.87	1	33.87	0.7610	0.3977
AC	242.11	1	242.11	5.44	0.0351
AD	278.89	1	278.89	6.27	0.0253
BC	91.20	1	91.20	2.05	0.1743
BD	77.44	1	77.44	1.74	0.2083
CD	65.61	1	65.61	1.47	0.2448
A^2^	1105.37	1	1105.37	24.83	0.0002
B^2^	443.00	1	443.00	9.95	0.0070
C^2^	940.03	1	940.03	21.12	0.0004
D^2^	2044.42	1	2044.42	45.93	< 0.0001
Residual	623.17	14	44.51		
Lack of Fit	470.62	10	47.06	1.23	0.4537
Pure error	152.55	4	38.14		
Cor Total	13,748.95	28			
R^2^	0.95		Predicted R^2^	0.78	
Adjusted R^2^	0.90		Adeq Precision	19.25	

The values of R^2^, adjusted R^2^, predicted R^2^, and adequacy precision were obtained as 0.95, 0.90, 0.78, and 19.25, respectively. According to Table 5, the P-values for the variables of PS concentration, *oak* dose, and contact time are all less than 0.05, indicating that these variables are statistically significant and have a significant effect on PS removal. As can be seen from Table 5, the p-value for C and BC, BD, and CD was greater than 0.05. This indicates that the results are not statistically significant, and these factors do not have a significant impact on the removal of PS. Whereas, the p-values for A^2^, B^2^, C^2^, D^2^, and AD were less than 0.01, indicating their significant effect on the removal of PS. In this model, the difference between R^2^ and predicted R^2^ was less than 0.2, which is considered to be good. In general, the concentration of PS, oak dose and contact time are the most important factors that affect the removal of PS by oak powder. The R-squared value is 0.95, indicating that the model explains 95% of the variability in PS removal^40^.

**Figure 4:**
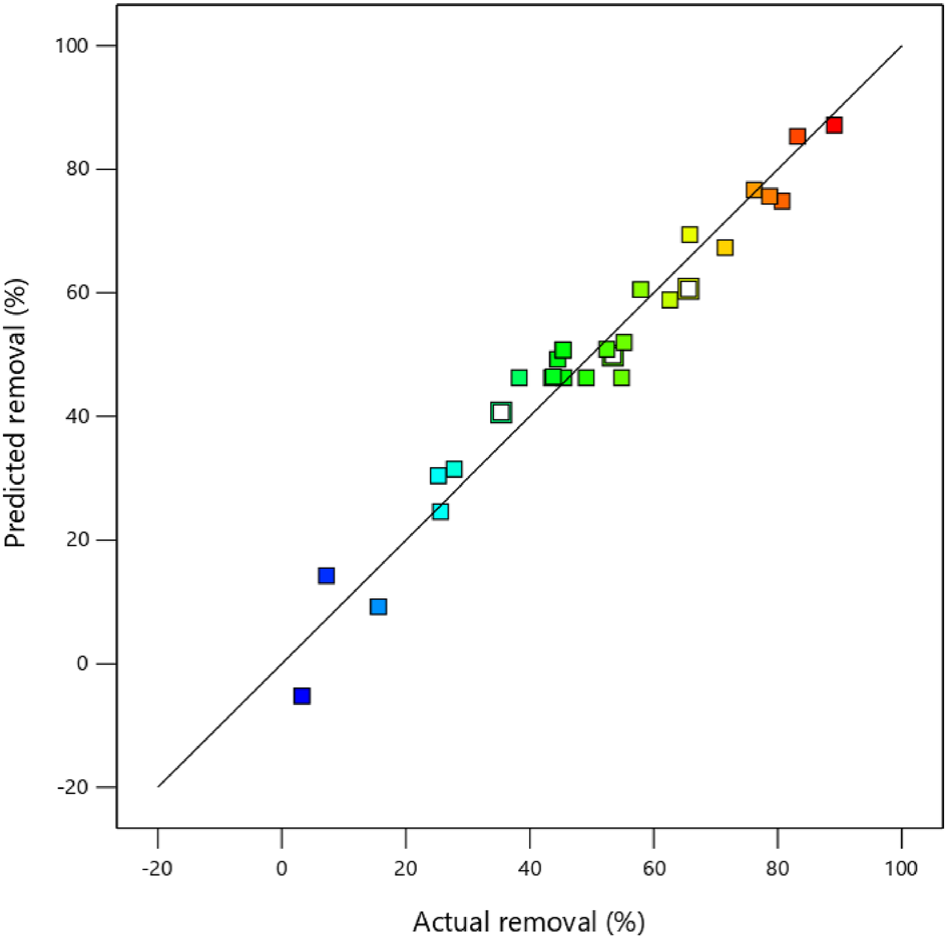
Distribution of experimental vs. predicted removal for PS by *oak* powder.

Figure 4 displays a comparison between the actual removal and the predicted removal. According to Fig. 4, it is evident that the model is adequate in providing a good prediction for PS removal.

### Effect of main factors on removal efficiency

**Figure 5:**
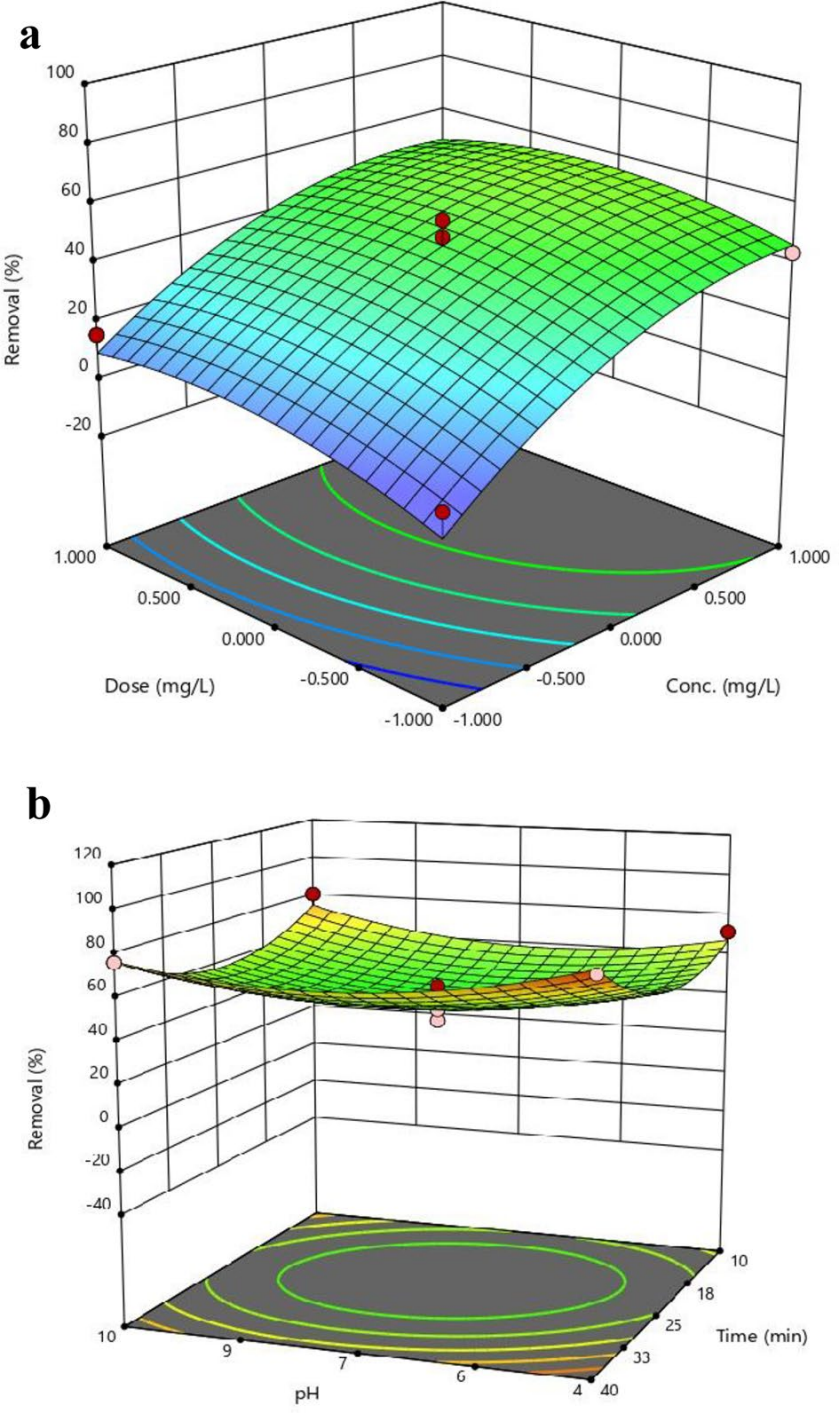
Response surface plots illustrating the effects of dose vs. concentration (**a**) and pH vs. time (**b**), generated using Design Expert 13 software.

The impact of significant variables, including PS concentration, oak dosage, pH, and contact time, on the removal efficiency was drawn by Design Expert 13 software, and the findings are illustrated in Fig. 5.

It is important to note that in order to explain the impact of one parameter on a response, other factors must be held constant at a zero level. For example, when the contact time variable increases from level 1 to + 1, the other variables such as PS concentration, oak dose, and pH remain constant at the zero level.

To calculate the removal efficiency of PS, *oak* powder was used with doses ranging from 100 to 400 mg/L. According to Fig. 5a, it can be observed that the removal efficiency increases as the *oak* dose increases. At low doses, *oak* particles form small and unstable flocs with PS, which reduces the efficiency of removal^41^. By increasing the dose of *oak*, the flocs come together with sufficient strength and create a larger surface area per unit volume of microplastic particles^42^. This causes PS to be adsorbed through intermolecular or van der Waals attraction^43^. With the bridging formation between the oak and PS particles, the flocs become heavier and increase sedimentation^44^. It should be noted that high doses of oak can cause the flocs to become heavy and break apart, leading to the redispersion of particles. As a result, the removal efficiency may decrease^41^. In the study conducted by YZhang et al., the removal efficiency improved from 20.1 to 70.7% when the coagulant dose of Mg (OH)_2_ was increased from 50 to 100 mg/L. Finally, by increasing the dose to 200 mg/L, the adsorption efficiency can be improved to 92.1%. However, with a further increase in Mg(OH)_2_ dosage up to 250 mg/L, the removal efficiency decreased by 10%^45^. In another study conducted by Yingying Duan et al., the PET removal efficiency increased from 50.67% to 92.05% as the coagulant dose was increased from 0 to 100 mg/L. When the coagulant dose was increased to 140 mg/L, the removal efficiency reached 84%^46^.

One important parameter for increasing removal efficiency during the coagulation process is the initial concentration of PS. According to Fig. 5a, the removal efficiency significantly increases as the initial concentration of PS. According to the results obtained, as the concentration of PS, the likelihood of collision with the coagulant also increases. Conversely, when the concentration of microplastic particles is low, the probability of collision decreases. The positive ions on the surface of the oak particles undergo hydrolysis, leading to compression of the bilayer structure of PS and a reduction in the repulsive force between particles of the same charge. As a result, the likelihood of collision between microplastics and coagulants increases. This process causes a significant portion of microplastics to lose stability and settle^47^. In another study, it was found that increasing the coagulant dose from 0 to 100 mg/L resulted in an increase in the removal efficiency of PET from 50.67 to 92.05%. However, at 140 mg/L, the removal efficiency initially increased and then decreased^32^.

From Fig. 5b, it is evident that the contact time has a direct impact on the removal efficiency. By increasing the time from 25 to 40 min, the removal efficiency improves by almost 14% (p-value < 0.05). By increasing the reaction time, microplastic particles have a greater opportunity to form flocs when in contact with oak particles, thereby enhancing the efficiency of PS removal. This factor can be seen as a sign of potential interaction between coagulants and PS microplastics. If coagulation is successful, the turbidity is effectively reduced through the settling of flocs^16^.

According to Fig. 5b, the removal efficiency decreased as the pH increased from 4 to 7, but then increased as the pH increased to 10 (p-value > 0.05). These observations can be related to the mechanism of surface charge neutralization. In fact, *oak* particles function as a natural polyelectrolyte. These particles have an opposite charge to that of microplastics and are typically attached to the surface of plastic particles. This attachment eventually neutralizes the surface charge^27,28^.

## Conclusion

In this research, *oak* powder were used as a natural, inexpensive, and eco-friendly coagulant. The Box-Behnken model was used to determine the optimal conditions for removal. FESEM, FTIR, and EDX analysis were used to characterize the properties of *oak* and PS particles. The FESEM image illustrates the impact of surface morphology and verifies the presence of PS particles accumulated on the surface of *oak* seed particles. The FTIR spectrum showed the bonds formed between PS and oak. EDX analysis determined the elements involved in the process of coagulation. The maximum PS microplastics (89.1%) was achieved by using *oak* powder under specific conditions. These conditions included an *oak* dose of 250 mg/L, a PS concentration of 900 mg/L, a contact time of 40 min, and a pH of 7. The results suggest that *oak* powder can effectively remove PS microplastics through surface adsorption and charge neutralization mechanisms, which are attributed to the presence of tannin compounds. Based on the results obtained, it has been found that the natural coagulant derived from *oak* powder possesses the ability to effectively compete with harmful chemical coagulants in removing microplastics from aqueous solutions.

## Data Availability

The datasets generated and analyzed during the current study were available from the corresponding author on reasonable request.
